# Active surveillance of carbapenem-resistant gram-negative bacteria to guide antibiotic therapy: a single-center prospective observational study

**DOI:** 10.1186/s13756-022-01103-0

**Published:** 2022-06-22

**Authors:** Qiqiang Liang, Juan Chen, Yongshan Xu, Yibing Chen, Man Huang

**Affiliations:** General Intensive Care Unit, The Second Affiliated Hospital, Zhejiang University School of Medicine, No.1511, Jianghong Road, Binjiang District, Hangzhou City, Zhejiang Province China

**Keywords:** Carbapenem-resistant gram-negative bacteria, Active surveillance, Empirical antibiotic therapy, Infection after colonization, Nosocomial infection prevention and control

## Abstract

**Background:**

Carbapenem-resistant gram-negative bacteria (CRGNB) have become a public health concern worldwide. The risk factors associated with CRGNB infection after colonization are unknown, nor is the optimal timing of antibiotic treatment, warranting further investigation.

**Methods:**

A 4-year single-center prospective observational study was conducted. CRGNB-colonized patients were incorporated on admission into our observation cohort for an active surveillance culture program, and analysis of risk factors associated with infections after CRGNB colonization was performed. We divided patients into empirical antibiotic therapy groups and standard antibiotic therapy groups according to whether antibiotics were used before or after cultures yielded a result to explore the relationship between the timing of antibiotics and clinical efficacy.

**Results:**

152 out of 451 CRGNB-colonized patients in the prospective observational cohort developed CRGNB infection. The risk factors associated with CRGNB infection after colonization included CRKP (*P* < 0.001, OR = 3.27) and CRPA (*P* < 0.001, OR = 2.97) colonization, history of carbapenems use (*P* < 0.001, OR = 5.48), and immunocompromise (*P* < 0.001, OR = 7.07). There were 88 infected patients in the empirical antibiotic therapy groups and 64 in standard antibiotic therapy groups. The mortality was lower in empirical therapy groups than standard therapy groups (17.0% vs. 37.5%, *P* = 0.004, OR = 0.32).

**Conclusions:**

CRGNB colonized patients who are prone to infection have some high-risk factors included CRKP and CRPA colonization, immunocompromise, and prior carbapenems use. Once infection occurs in CRGNB-colonized patients, early use of effective antibiotics may be associated with reduced mortality, but more studies are needed.

## Background

Carbapenem-resistant gram-negative bacteria (CRGNB) are one of the most serious multidrug-resistant bacteria (MDR) globally, accounting for a significant portion of Hospital Acquired Infections (HAI) and are associated with increased mortality and prolonged hospitalizations [1, 2]. Active surveillance for early recognition of CRGNB carriers and other infection control measures, including contact isolation and decontamination strategies, are essential to reduce the spread of MDR [3, 4]. We also found that active surveillance combined with early or preemptive isolation could reduce the spread of carbapenem-resistant enterobacteria (CRE) in clinical practice [5]. A meta-analysis of 10 observational studies demonstrated CRE-colonized patients had a 16.5% risk of infection. Recent studies have demonstrated that the infection rates after CRGNB colonization ranged from 11 to 30% and were influenced by CRGNB endemicity and the implementation of IPC strategies [6].

Stringent measures have been designed to block the spread of CRE infection, encompassing active surveillance, strict contact isolation and hand hygiene, selective digestive decontamination (SDD), and restricted use of carbapenem antibiotics [4, 7]. Antibiotics such as polymyxin B and ceftazidime-avibactam are acknowledged for their efficiency against CRGNB, while other measures have been developed, including the combination of antibiotics (double carbapenem combinations), prolonged infusion of carbapenem, and inhalation of polymyxin B and aminoglycosides to provide more options for treatment of CRGNB infections [8–12]. Nowadays, guidelines recommend early empirical antibiotic treatments for severe infections but tend to be cautious for the optimal antibiotic timing in CRGNB infection because of the paucity of effective antibiotics and potential inferior efficacy of the "old" antibiotics.

Antibiotics are usually indicated after CRGNB infection with documentation by culture yields, which may delay treatment for at least 2–3 days. Some studies have pointed out that earlier use of sensitive antibiotics may reduce the mortality of CRE bloodstream infection; however, there is a risk that indiscriminate use of antibiotics makes the pathogen more resistant, leading to antibiotic resistance [13]. Based on our experience, it seems more reasonable to use susceptibility-guided antibiotic treatment in CRGNB-colonized patients, but there is little empirical evidence. The purpose of this study was to explore the risk factors of CRGNB infection after colonization by active surveillance and to analyze the clinical effects of susceptibility-guided antibiotic treatment.

## Methods

### Study design

It was a single-center case control prospective observational study conducted at the General Intensive Care Unit (ICU) of the Second Affiliated Hospital of Zhejiang University, a 1200-bed tertiary academic care hospital with 40 intensive care beds in Hangzhou China. The study period spanned from 2017.01.01 to 2020.12.31. Since 2017.01, our ICU has implemented robust infection prevention and control (IPC) measures against CRE, emphasizing active surveillance combined with early or preemptive isolation. We have adopted active surveillance and individual isolation for patients with a high risk of MDR carriage, with two negative tests prerequisites for release from isolation [5]. In this study, CRGNB-colonized patients in active surveillance at admission were incorporated into the observation cohort. The purpose was to study the high-risk factors of infection after colonization of CRGNB in the first phase of the study. Therefore, the observation subjects were all patients with CRGNB colonization at active surveillance by collecting the risk factors of exposure, and the outcome was the occurrence of CRGNB infection. The purpose of the second phase of the study is to explore whether active surveillance of CRGNB has a positive impact on the use of antibiotics. The subjects were all patients with secondary CRGNB infection. The timing and clinical prognosis of antibiotic use were observed, and the outcome was prognosis such as transferred out of ICU or died. This study was conducted after approval from the ethics committee of the Second Affiliated Hospital of Zhejiang University school of medicine (Batch number IRB-2016-1511).

### Data definition

#### Inclusion criteria

Patients who underwent active surveillance on admission with any positive surveillance culture from the throat, rectal, or inguinal swabs, or other specimens sent by the clinicians. Inclusion criteria for active surveillance:

1. Patients transferred from other medical institutions, including (a) Patients hospitalized for more than seven days; (b) Patients hospitalized for less than seven days but who were in contact with suspected CRE patients or symptomatic; (c) Patients with prolonged hospitalization in private rehabilitation hospitals and nursing homes; (d) CRE-infected patients who were transferred from general wards but were hospitalized for less than 3 days.
2. Symptomatic patients transferred from high-risk general wards (a high-risk ward was defined as a ward with patients suffering from CRE infection within the preceding three months according to the hospital antimicrobial resistance monitoring report);
3. Patients who had been confirmed to be CRE carriers within the past year.

#### Exclusion criteria

Diagnosis of CRGNB infection on admission, admission less than 48 h, and age under 16. Complicated CRGNB mixed infection. If the secondary pathogenic bacteria and colonized bacteria are different types of CRGNB, we don’t included in the analysis.

#### Subgroup definition

Empirically sensitive antibiotic therapy was given to CRGNB-colonized patients diagnosed with CRGNB infection, and before the culture yielded a result, antibiotic selection (ceftazidime-avibactam, colistin, and tigecycline) was based on the previous (colonization) CRGNB susceptibility results. Standard antibiotic therapy groups were given to CRGNB-colonized patients diagnosed with CRGNB infection, and antibiotic selection was based on the CRGNB susceptibility results after the cultures yielded results.

#### Infection definition

Diagnosis of CRGNB infection was made by two senior attending physicians independently. Diagnosis of Bloodstream infection was made in the presence of clinical manifestations in patients with positive blood cultures. Diagnosis of Hospital-Acquired Pneumonia and Urinary tract infection was based on the 2016 USA IDS/ATS guidelines [14] and on the 2009 USA IDS guidelines [15]. Intra-abdominal infection and infectious diarrhea was used 2009 USA SIS/IDSA guidelines and 2017 USA IDSA guidelines [16, 17]. Gastrointestinal infection included intra-abdominal infection and infectious diarrhea. Other rare infections, including skin and soft tissue infection and intracranial infection, also have guidelines for reference [18, 19].

#### Other important definitions

Medication history was limited to 3 months before CRGNB colonization, including cephalosporins, carbapenems, oral or intravenous glucocorticoids. Invasive procedures included catheterizations of deep veins and arteries for continuous renal replacement therapy, pulse indicator continuous cardiac output, and extracorporeal membrane oxygenation. Immunocompromised patients included patients with chemotherapy and long-term use of glucocorticoids or immunosuppressants. Other department history refers to patients who were admitted to other departments for more than 3 days. Other hospital history refers to the hospitalization in other hospitals or nursing institutions in the past six months. The definition of Acute kidney injury was based on the guidelines of The Kidney Disease: Improving Global Outcomes [20].

### Microbiological tests

CRGNB strains including CRE, carbapenem-resistant *Klebsiella pneumoniae* (CRKP), carbapenem-resistant *Pseudomonas aeruginosa* (CRPA), and carbapenem-resistant *Acinetobacter baumannii* (CRAB), were resistant to at least one of the carbapenems, including imipenem, meropenem, and ertapenem. The Antimicrobial susceptibility tests were performed with automated microbial identification and drug susceptibility systems (VITEK2 AST-GN16 France). Minimum inhibitory concentration (MIC) determination and interpretation complied with standards established by the Clinical and Laboratory Standards Institute (CLSI). Resistance to carbapenem was defined as a MIC ≥ 2 mg/L for imipenem or meropenem according to CLSI guidelines.

### Statistical methods

All statistical analyses were performed in R and RStudio. In univariate analysis, numerical variables were tested by independent sample T-test, dichotomous variables were compared by chi-square test, and a *P* value less than 0.05 was considered statistically significant. We include all the variables with *P* < 0.15 in univariate analysis into multivariate analysis in order to avoid omitting possible variables. Odds ratio (OR) and 95% confidence interval (CI) were calculated to evaluate the strength of association.

## Results

**Fig. 1:**
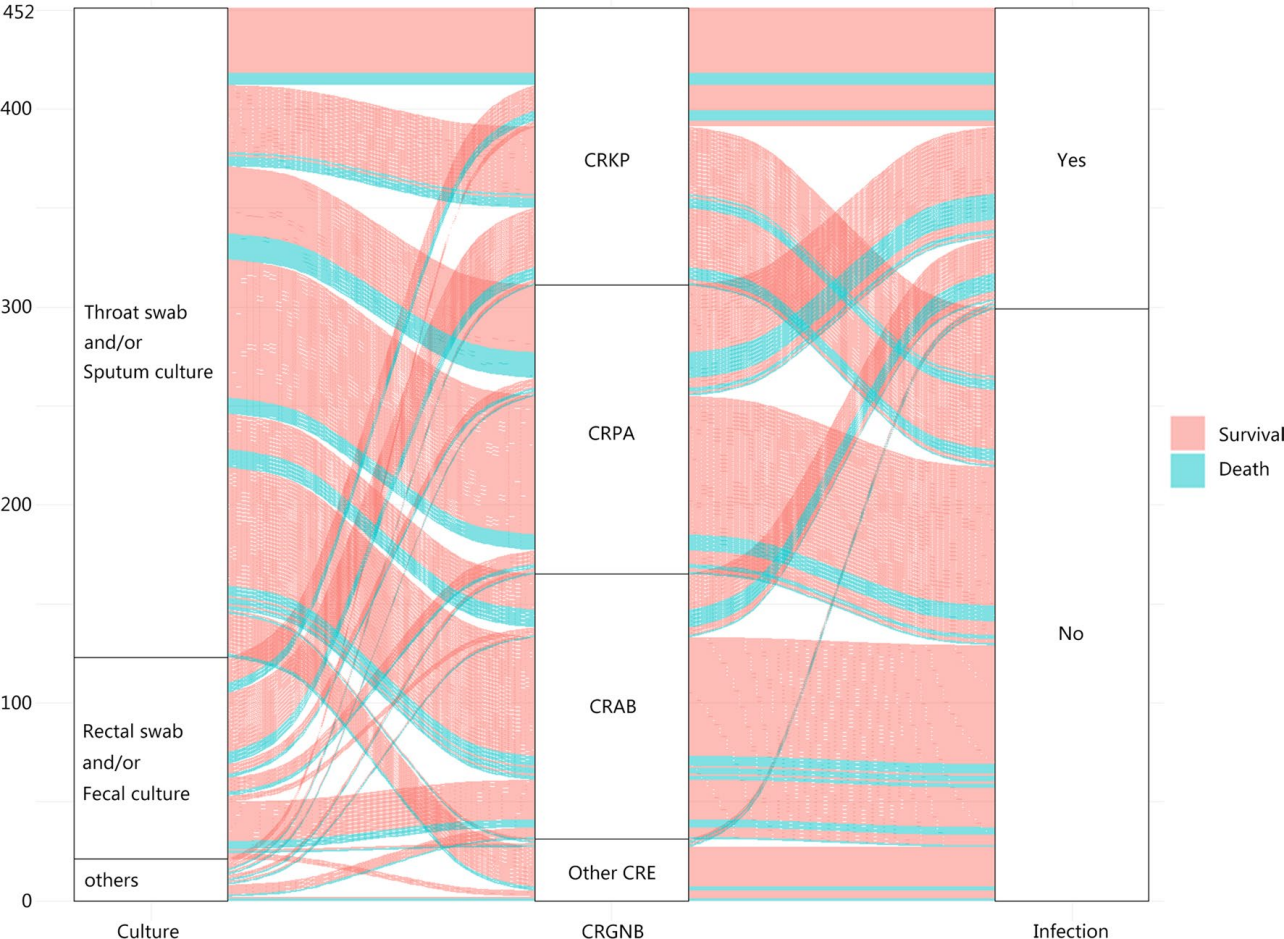
Alluvial diagram of the feature distribution in CRGNB colonization patients by active surveillance

From 2017.01.01 to 2020.12.31, our center treated a total of 6645 patients with 3754 cases that underwent active surveillance. 548 (14.6%) patients yielded positive CRGNB surveillance cultures, and 97 cases presented with CRGNB infection on admission. Finally, 451 (12.0%) colonized patients were included, among which 152 (33.7%) developed CRGNB infection. There was no significant difference in gender, age, primary disease, acute physiology and chronic health evaluation (APACHE) II scores, and distribution of surveillance swab sites between the CRGNB infection and colonization groups in Table 1. Surveillance cultures with the greatest proportion of positive yields came from throat swabs and sputum, followed by rectal swabs, inguinal swabs, and feces. Interestingly, the proportion of CRKP and CRPA was higher in the infection group, while CRAB was more common in the colonization group. The relationship between the distribution of swab sites, bacterial type, infection or not, or colonization and outcome is shown in Fig. 1. Compared with the colonization group, infected patients were associated with prior use of carbapenems (46.7% vs. 14.7%, *P* < 0.001, OR = 5.08. 95%CI 3.23–7.97) and glucocorticoids (28.9% vs. 15.7%, *P* = 0.001, OR = 2.18, 95%CI 1.36–3.49) use, and invasive procedures (87.5% vs. 76.3%, *P* = 0.005, OR = 2.18, 95%CI 1.26–3.78). Moreover, patients in the infection group were more likely to be immunocompromised (47.4% vs. 23.1%, *P* < 0.001, OR = 3.00, 95%CI 1.98–4.55), previously hospitalized in another center (59.2% vs. 49.2%, *P* = 0.043, OR = 1.52, 95%CI 1.01–2.28) and previously admitted in another department (34.2% vs. 22.4%, *P* = 0.007, OR = 1.80, 95%CI 1.17–2.77) than the colonization group. The incidence of septic shock (55.2% vs. 27.1%, *P* < 0.001, OR = 3.32, 95%CI 2.21–5.00) and AKI (48.7% vs. 23.1%, *P* < 0.001, OR = 3.16, 95%CI 2.08–4.79) were higher in the infection group, with longer ICU stay, hospital stay and higher mortality (25.6% vs. 14.0%, *P* = 0.002, OR = 2.11, 95%CI 1.30–3.44). See Table 1 for more details.

**Table 1:** Baseline characteristic of the CRGNB colonized patients at active surveillance

	Infection after colonization, N = 152	Colonization, N = 299	*P*	OR (95%CI)
Age, year, mean (SD)	60.6 (12.4)	59.0 (11.9)	0.32	/
Male sex, no. (%)	112 (73.6%)	206 (68.9%)	0.29	0.85 (0.51–1.22)
Primary disease (%)				
Cerebrovascular accidents, no. (%)	38 (25.0%)	79 (26.4%)	0.74	0.93 (0.59–1.45)
Cardiac insufficiency, no. (%)	16 (10.5%)	26 (8.7%)	0.52	1.24 (0.64–2.38)
Trauma, no. (%)	61 (40.1%)	115 (38.5%)	0.75	1.07 (0.72–1.59)
Infection, no. (%)	49 (32.2%)	82 (27.4%)	0.28	1.25 (0.82–1.92)
Malignant tumor, no. (%)	11 (7.2%)	26 (8.7%)	0.59	0.82 (0.39–1.71)
Diabetes mellitus, no. (%)	14 (9.2%)	41 (13.7%)	0.17	0.64 (0.34–1.21)
APACHE II scores, median (IQR)	16.8 (7.5–25.6)	16.4 (8.0–29)	0.29	/
Intubation or tracheotomy, no. (%)	76 (50.0%)	162 (54.2%)	0.40	0.86 (0.57–1.25)
Prior cephalosporins history, no. (%)	116 (76.35)	198 (66.2%)	0.028	1.64 (1.05–2.56)
Prior carbapenems history, no. (%)	71 (46.7%)	44 (14.7%)	< 0.001	5.08 (3.23–7.97)
Invasive operation, no. (%)	133 (87.5%)	228 (76.3%)	0.005	2.18 (1.26–3.78)
Operation History, no. (%)	20 (13.2%)	29 (9.7%)	0.26	1.41 (0.76–2.58)
Glucocorticoid history, no. (%)	44 (28.9%)	47 (15.7%)	0.001	2.18 (1.36–3.49)
Immunocompromise, no. (%)	72 (47.4%)	69 (23.1%)	< 0.001	3.00 (1.98–4.55)
Other hospital history, no. (%)	90 (59.2%)	147 (49.2%)	0.043	1.52 (1.01–2.28)
Another department history, no. (%)	52 (34.2%)	67 (22.4%)	0.007	1.80 (1.17–2.77)
CRGNB type, no. (%)				
CRKP	60 (39.5%)	80 (26.7%)	0.006	1.78 (1.18–2.70)
CRPA	56 (36.8%)	90 (30.1%)	0.15	1.35 (0.89–2.04)
CRAB	32 (21.1%)	102 (34.1%)	0.004	0.51 (0.32–0.84)
Other CRE	4 (2.6%)	27 (9.0%)	0.025	0.34 (0.12–0.90)
MIC of carbapenems (mg/L)				
Imipenem median (IQR)	16 (8–64)	16 (8–64)	1.0	/
Ertapenem median (IQR)	8 (4–32)	8 (4–32)	1.0	/
Active surveillance sites, no. (%)				
Throat swab and/or sputum culture	115 (75.6%)	213 (71.2%)	0.31	1.25 (0.80–1.96)
Rectal swab and/or fecal culture	30 (19.7%)	72 (24.1%)	0.29	0.77 (0.48–1.25)
Other cultures	7 (4.6%)	14 (4.7%)	0.97	0.98 (0.38–2.48)
Septic shock, no. (%)	84 (55.2%)	81 (27.1%)	< 0.001	3.32 (2.21–5.00)
Acute kidney injury, no. (%)	74 (48.7%)	69 (23.1%)	< 0.001	3.16 (2.08–4.79)
Death, no. (%)	39 (25.6%)	42 (14.0%)	0.002	2.11 (1.30–3.44)
Length of ICU stay, day, median (IQR)	17.8 (3.0–34.5)	8.9 (2.5–30.0)	< 0.001	/
Length of hospital stay, day, median (IQR)	27.6 (6.5–65.5)	19.3 (5.0–45.0)	< 0.001	/

CRGNB: carbapenem-resistant Gram-negative bacteria; CRKP: carbapenem-resistant *Klebsiella pneumoniae*; CRAB: carbapenem-resistant *Acinetobacter baumannii;* CRPA: carbapenem-resistant *Pseudomonas aeruginosa*; CRE: carbapenem-resistant enterobacteria; SD: standard deviation; APACHE II: acute physiology and chronic health evaluation; IQR: interquartile range; ICU: intensive care unit; OR: odds ratio; CI: confidence interval

Furthermore, we identified six risk factors of CRGNB infection after colonization by multiple regression analysis, including CRKP colonization (*P* < 0.001, OR = 3.27, 95%CI 1.80–5.95), CRPA colonization (*P* < 0.001, OR = 2.97, 95%CI 1.63–5.40), prior admission in other departments (*P* = 0.029, OR = 1.78, 95%CI 1.06–2.97), invasive procedure (*P* < 0.001, OR = 6.22, 95%CI 2.57–15.03), history of carbapenem use (*P* < 0.001, OR = 5.48, 95%CI 3.27–9.19), and immunocompromise (*P* < 0.001, OR = 7.07, 95%CI 3.90–12.80). More details are shown in Table 2.

**Table 2:** High-risk factors of infection after colonization in multivariate regression analysis

Variable	*P*	OR (95%CI)
CRKP colonization	< 0.001	3.27 (1.80–5.95)
CRPA colonization	< 0.001	2.97 (1.63–5.40)
Another department history	0.029	1.78 (1.06–2.97)
Invasive operation	< 0.001	6.22 (2.57–15.03)
Carbapenems history	< 0.001	5.48 (3.27–9.19)
Immunocompromise	< 0.001	7.07 (3.90–12.80)

CRKP: carbapenem-resistant *Klebsiella pneumoniae*; CRPA: carbapenem-resistant *Pseudomonas aeruginosa*; OR: odds ratio; CI: confidence interval

**Fig. 2:**
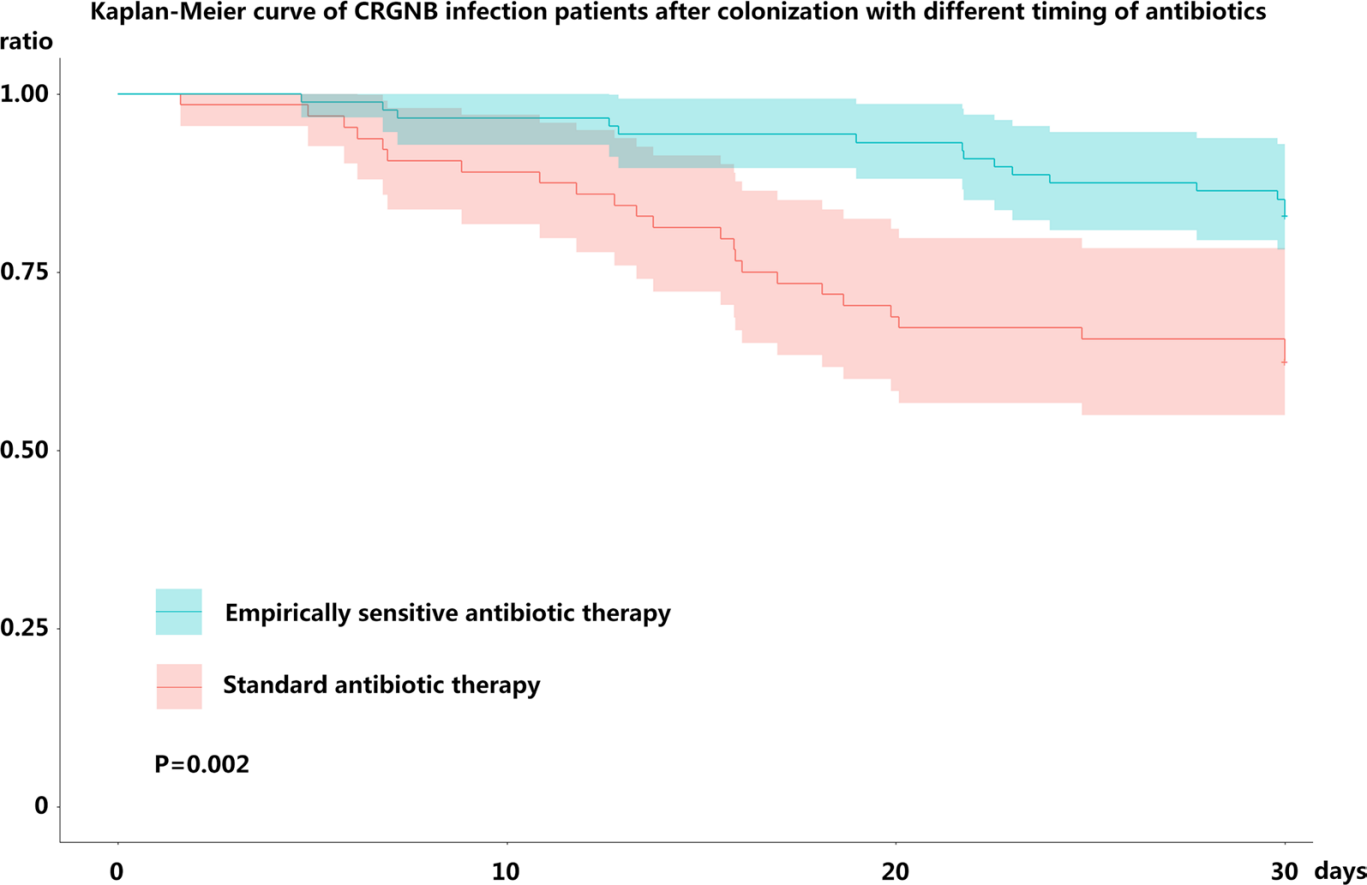
Kaplan–Meier curve of CRGNB infection patients after colonization with different timing of antibiotics

Patients infected after colonization were divided into empirically sensitive antibiotic therapy groups (n = 88) and standard antibiotic therapy groups (n = 64), depending on the timing of antibiotic use. No statistical difference in basic characteristics was found between the two groups (Table3). The incidence of bloodstream infection was higher in empirically therapy groups than standard therapy groups; however, no statistical differences were found for the remaining part of the infection and the bacterial types. The most common antibiotic therapy used was combination therapy based on tigecycline and colistin, followed by ceftazidime–avibactam monotherapy. The proportion of combinations with polymyxin B was similar (15.9% vs. 15.6%, *P* = 0.96) and ceftazidime–avibactam monotherapy was more common in the empirically sensitive antibiotic therapy groups than in the standard antibiotic therapy groups (14.7% vs. 6.2%, *P* = 0.1). There was no statistical difference in the distribution of antibiotics regimens for CRGNB between the two groups which means that the difference in mortality did not cause by use of specific potent antibiotics. The average interval between colonization and infection was around 13 days, and ICU hospitalization was about 16 days after CRGNB infection. The mortality was lower in the empirical therapy groups than standard therapy groups (17.0% vs. 37.5%, *P* = 0.004, OR = 0.32, 95%CI 0.16–0.73), which was substantiated by survival curve analysis (*P* = 0.002) (Fig. 2).

**Table 3:** The timing and clinical efficacy of antibiotics for infected patients after colonization

	Empirically sensitive antibiotic therapy groups, N = 88	Standard antibiotic therapy groups, N = 64	*P*	OR (95%CI)
Age-year, mean (SD)	61.2 (9.8)	59.8 (10.3)	0.60	/
Male sex, no. (%)	66 (75.0%)	46 (71.8%)	0.66	0.85 (0.41–1.76)
APACHE II scores, median (IQR)	17.0 (8.0–28.0)	16.4 (7.0–32.0)	0.20	/
Cerebrovascular accidents, no. (%)	22 (25.0%)	16 (25.0%)	1.00	1.00 (0.47–2.10)
Cardiac insufficiency, no. (%)	9 (10.2%)	7 (10.9%)	0.88	0.93 (0.32–2.63)
Trauma, no. (%)	38 (43.2%)	23 (35.9%)	0.36	1.35 (0.69–2.62)
Infection, no. (%)	24 (27.3%)	25 (39.1%)	0.12	0.58 (0.29–1.16)
Malignant tumor, no. (%)	9 (10.2%)	2 (3.1%)	0.09	3.52 (0.73–16.94)
Diabetes mellitus, no. (%)	9 (10.2%)	5 (7.8%)	0.61	1.34 (0.42–4.22)
Intubation or tracheotomy, no. (%)	45 (51.1%)	31 (48.4%)	0.74	1.11 (0.58–2.12)
Invasive operation, no. (%)	72 (81.8%)	51 (79.6%)	0.74	1.14 (0.51–2.59)
Operation History, no. (%)	10 (11.3%)	10 (15.6%)	0.44	0.69 (0.27–1.77)
Immunocompromise, no. (%)	46 (52.2%)	26 (40.6%)	0.15	1.60 (0.83–3.06)
Septic shock, no. (%)	44 (50.0%)	40 (62.5%)	0.12	0.60 (0.31–1.15)
Acute kidney injury, no. (%)	40 (45.5%)	34 (53.1%)	0.35	0.73 (0.38–1.40)
Infection sites, no. (%)				
Pulmonary infection	64 (72.7%)	50 (78.1%)	0.45	0.74 (0.35–1.59)
Bloodstream infection	35 (39.8%)	10 (15.6%)	0.001	3.56 (1.60–7.92)
Urinary tract infection	26 (29.5%)	16 (25.0%)	0.53	1.25 (0.61–2.60)
Gastrointestinal infection	21 (23.9%)	16 (25.0%)	0.87	0.94 (0.45–1.99)
Other sites infections	37 (42.0%)	25 (39.1%)	0.71	1.13 (0.58–2.18)
CRGNB types, no. (%)				
CRKP	38 (43.2%)	22 (34.4%)	0.27	1.45 (0.74–2.82)
CRPA	31 (35.2%)	25 (39.1%)	0.63	0.84 (0.43–1.65)
CRAB	17 (19.3%)	15 (23.4%)	0.54	0.78 (0.35–1.71)
Other CRE	2 (2.3%)	2 (3.1%)	1.00	0.72 (0.10–5.25)
Antibiotic therapy, no. (%)				
Combinations with Tigecycline	25 (28.4%)	14 (21.8%)	0.36	1.41 (0.67–3.01)
Combinations with Polymyxin B	14 (15.9%)	10 (15.6%)	0.96	1.02 (0.42–2.47)
Ceftazidime–avibactam monotherapy	13 (14.7%)	4 (6.2%)	0.10	2.60 (0.81–8.39)
Other therapies	36 (40.9%)	36 (56.3%)	0.06	0.53 (0.28–1.03)
Timing of antibiotic intervention, hours, median (IQR)	8.4 (3.6–17.2)	45.6 (15.4–69.4)	0.001	/
Duration from colonization to infection, day, median (IQR)	13.5 (4.0–19.0)	12.8 (4.0–17.5)	0.54	/
Length of ICU stay after infection, day, median (IQR)	16.7 (7.5–32.5)	16.8 (6.0–35.5)	0.61	/
Length of hospital stay after infection, day, median (IQR)	32.8 (10.5–75.0)	24.2 (6.0–65.0)	0.22	/
Death, no. (%)	15 (17.0%)	24 (37.5%)	0.004	0.34 (0.16–0.73)

CRGNB: carbapenem-resistant gram-negative bacteria; CRKP: carbapenem-resistant *Klebsiella pneumoniae*; CRAB: carbapenem-resistant *Acinetobacter baumannii;* CRPA: carbapenem-resistant *Pseudomonas aeruginosa*; CRE: carbapenem-resistant enterobacteria; SD: standard deviation; APACHE II: acute physiology and chronic health evaluation; IQR: interquartile range; ICU: intensive care unit; OR: odds ratio; CI: confidence interval

## Discussion

Nearly one third of CRGNB colonized patients develop secondary infections who underwent active surveillance on ICU admission, which is a very high proportion. These patients may benefit from early use of empirically sensitive antibiotic therapy.

Contact isolation and hand hygiene have been widely emphasized, while much controversy surrounds the implementation of active surveillance for its questionable cost-effectiveness [21]. CRGNB colonization has increased with the widespread prevalence of CRGNB in Asia. Indeed, at clinical level, the medical personnel are often overwhelmed by the concomitant increase in workload following the implementation of basic IPC measures. In some areas with limited resources, active surveillance is not even recommended for asymptomatic patients [21].

Clinical studies have consistently shown that CRGNB colonization is a high-risk factor for infection [22, 23]. However, the risk factors of CRGNB infection after colonization are not necessarily the same for CRGNB colonization which may also depend on the patient's immune status. It is widely acknowledged that advanced age, serious disease status, including higher sequential organ failure assessment (SOFA), Pitt score, Charlson score, carbapenem drug exposure, and invasive catheterization are high-risk factors for MDR infection after colonization [23, 24].

For CRGNB-colonized patients, a series of stringent measures were adopted, including contact isolation and strict hand hygiene to avoid cross-infection; nonetheless, CRGNB infection after colonization was unavoidable, leading to a poor patient prognosis. Addressing risk factors of infection after colonization earlier may potentially be the solution to this conundrum. Herein, CRKP and CRPA colonization were associated with greater CRGNB infection rates than CRAB and other CRE. Other identified risk factors included invasive catheterization, history of carbapenem use, and immunocompromise. It is important to note that the high-risk factors analysis does not accurately predict which patients will develop CRGNB infection. In this regard, many scoring systems based on high-risk factors have been designed to solve this problem. However, no consensus has been reached on the accuracy of these scoring systems due to the inherent differences in regional characteristics, medical habits, and types of diseases [23–25]. Building a more efficient and robust prediction model by artificial intelligence may potentially be the solution. SDD programs have been implemented in some European countries to curb CRGNB infection rates after CRE colonization; however, the efficiency is uncertain due to their implementation in low CRE epidemic areas [21]. Given the uncertainty of the effects of SDD, our center did not adopt any decontamination strategy.

An increasing body of evidence suggests that early empirical antibiotic use can reduce mortality of severe sepsis or severe pneumonia [26, 27]. The Infectious Diseases Society of America (IDSA) recommends that empirical antibiotics use should take it into account of antibiotic sensitivity data in the past six months and antibiotic exposure in the past 30 days [2]. The European consensus recommends that the empirical treatment of CRGNB should be limited to critically ill patients, and it is necessary to know the drug sensitivity of CRGNB to carry out empirical treatment [13]. In addition, misuse of antibiotics can also be dangerous. Elena Carrara et al. pointed out that inappropriate empirical antibiotic use was closely related to mortality MDR strain infections [28].

Interestingly, in a randomized controlled study by Yael Zak-Doron et al. the mortality rate was not reduced in patients with severe CRGNB infection that received early (during the first 48 h after culture taking) empirical antibiotic treatments with mainly colistin (96%) [29]. This conclusion is surprising since 77% of CRGNB infections were related to CRAB, and all patients received empirical antibiotic treatment. If empirical treatment can prevent early death, this exclusion may bias the results to be ineffective, but unfortunately, the study does not have complete data on early death and its empirical treatment [29]. In addition, the study found that documented colonization (*P* = 0.044, OR = 0.76) may be a potential protective factor, which may be related to empirical antibiotic therapy [29]. In appropriate empirical treatment is dangerous and emphasizes the importance of active surveillance. Active surveillance provides more background information on bacterial colonization, although subsequent infections in CRGNB colonized patients are possibly mixed infections or another pathogenic bacterium. However, during severe infection or even septic shock management, microbiological data from CRGNB-colonized patients provides evidence of the potential etiology with drug sensitivity results to help clinicians in the decision-making process [13]. Herein, the empirical antibiotic treatments of tigecycline, colistin, ceftazidime–avibactam achieved good results. We recommend that secondary infected patients with CRGNB colonization use of directed empirical antibiotics when new infections are uncontrollable, but we need to avoided abuse according to the drug resistance, virulence, and invasive site of local prevalence of drug-resistant bacteria.

There were some limitations in our research. This study was a single-center prospective observational study, and the level of evidence was low. To substantiate our findings, more studies should be conducted involving multiple centers and clinical trials. Moreover, potential confounding factors such as changes in recommendations for CRGNB treatment and clinical experience of physicians were not considered.

## Conclusions

CRGNB colonized patients who are prone to infection have some high-risk factors included CRKP and CRPA colonization, immunocompromise, and prior carbapenems use. For patients with new infections after CRGNB colonization, early use of effective antibiotics may be associated with reduced mortality, but more studies are needed.

## Data Availability

The data that support the findings of this study are available from Man Huang but restrictions apply to the availability of these data, which were used under license for the current study, and so are not publicly available.

## Abbreviations

CRGNB: Carbapenem-resistant gram-negative bacteria
CRE: Carbapenem-resistant enterobacteria
ICU: Intensive care unit
IPC: Infection prevention and control
MDR: Multiple drug resistance
CRKP: Carbapenem-resistant *Klebsiella pneumoniae*
CRPA: Carbapenem-resistant *Pseudomonas aeruginosa*
CRAB: Carbapenem-resistant *Acinetobacter baumannii*
MIC: Minimum inhibitory concentration
CLSI: Clinical and laboratory standards institute
OR: Odds ratio
CI: Confidence interval
APACHE: Acute physiology and chronic health evaluation
SOFA: Sequential organ failure assessment
SDD: Selective digestive decontamination
